# Epidemiology of Human Mpox — Worldwide, 2018–2021

**DOI:** 10.15585/mmwr.mm7203a4

**Published:** 2023-01-20

**Authors:** Andrea M. McCollum, Victoria Shelus, Alexandra Hill, Tieble Traore, Bernard Onoja, Yoshinori Nakazawa, Jeffrey B. Doty, Adesola Yinka-Ogunleye, Brett W. Petersen, Christina L. Hutson, Rosamund Lewis

**Affiliations:** ^1^Division of High-Consequence Pathogens and Pathology, National Center for Emerging and Zoonotic Infectious Diseases, CDC; ^2^Epidemic Intelligence Service, CDC; ^3^Health Emergencies Programme, World Health Organization, Geneva, Switzerland.

Monkeypox (mpox) is a zoonotic disease caused by *Monkeypox virus* (MPXV), an *Orthopoxvirus*; the wild mammalian reservoir species is not known. There are two genetic clades of MPXV: clade I and clade II (historically found in central and west Africa, respectively), with only Cameroon reporting both clades (*1*). Human cases have historically been reported from 1) mostly rural, forested areas in some central and west African countries; 2) countries reporting cases related to population migration or travel of infected persons; and 3) exposure to imported infected mammals (*2*). The annual number of cases in Africa has risen since 2014 and cumulatively surpassed reports from the previous 40 years for most countries. This reemergence of mpox might be due to a combination of environmental and ecological changes, animal or human movement, the cessation of routine smallpox vaccination since its eradication in 1980, improvements in disease detection and diagnosis, and genetic changes in the virus (*2*). This report describes the epidemiology of mpox since 1970 and during 2018–2021, using data from national surveillance programs, World Health Organization (WHO) bulletins, and case reports, and addresses current diagnostic and treatment challenges in countries with endemic disease. During 2018–2021, human cases were recognized and confirmed in six African countries, with most detected in the Democratic Republic of the Congo (DRC) and Nigeria. The reemergence and increase in cases resulted in its being listed in 2019 as a priority disease for immediate and routine reporting through the Integrated Disease Surveillance and Response strategy in the WHO African region.* In eight instances, patients with mpox were identified in four countries outside of Africa after travel from Nigeria. Since 2018, introductory and intermediate training courses on prevention and control of mpox for public health and health care providers have been available online at OpenWHO.^†^^,^^§^ The global outbreak that began in May 2022^¶^ has further highlighted the need for improvements in laboratory-based surveillance and access to treatments and vaccines to prevent and contain the infection, including in areas of Africa with endemic mpox.

Annual mpox case and death counts during 2018–2021 were compiled from national surveillance data, WHO bulletins, and published case reports or outbreak investigations, and were verified with country surveillance teams; these data are presented with human mpox case report data since 1970. Since 2018, cases occurred in six African countries: Cameroon, Central African Republic (CAR), DRC, Nigeria, Republic of the Congo (ROC), and Sierra Leone (Table 1) (Figure). DRC reported >3,000 suspected cases per year, with a peak of 6,216 cases and 222 deaths in 2020. During 2018–2021, the number of confirmed mpox cases in CAR (79) from seven localities represented a notable increase compared with previous years, and an average of nine annual mpox outbreaks have occurred in CAR since 2018. In addition, nine cases were confirmed in Cameroon, where no human case of mpox had been documented since 1989; in a 2018 case, the virus shared genetic similarity with a clade II strain previously isolated from Nigeria (*1*), and additional cases were reported in different regions of the country in 2020 and 2021. Two cases each in ROC and Sierra Leone were reported during 2018–2021.

**TABLE 1:** Reported suspected and confirmed cases of human mpox and mpox-related deaths, by country — Africa, 1970–2021

Country	Year	Location	Suspected cases	Confirmed cases*	Deaths
**Benin^†^**	1978	Parakou	NA	1	0
**Cameroon**	1979	Mfou	NA	1	0
	1989	Nkoteng	NA	1	0
	2018	Akwaya, Njikwa	NA	2	0
	2019	Ekondo Titi	NA	1	0
	2020	Ayos, Doumé	NA	2	0
	2021	Ayos, Nkambé	NA	4	0
**Central African Republic**	1984	Sangha	NA	6	0
	2001	Mbomou	0	3	2
	2010	Lobaye	0	1	0
	2012	Ouham	0	2	0
	2015	Haute Kotto, Mbomou	3	4	4
	2016	Basse Kotto, Mbomou	7	4	2
	2017	Lobaye, Mbomou	1	6	0
	2018	Lobaye, Mbomou, M’Poko, Ombella	5	28	0
	2019	Lobaye, Ouaka	18	15	2
	2020	Lobaye, Mbomou, Sangha Mbaéré	2	8	0
	2021	Haute-Kotto, Lobaye, Mambéré Kadéi, Mbomou, Sangha Mbaéré	25	28	2
**Côte d’Ivoire**	1971	Abengourou	NA	1	0
	1981	Daloa	NA	1	NA
**Democratic Republic of the Congo**	1970–1986	Multiple provinces	NA	386	NA
	1987–1995		NA	NA	NA
	1996–2004		>200 per year	NA	NA
	2005–2015		>1,000 per year	NA	NA
	2016		3,750	NA	NA
	2017		2,500	NA	NA
	2018		3,784	NA	78^§^
	2019		5,288	NA	107^§^
	2020		6,216	NA	222^§^
	2021		2,841	NA	76^§^
**Gabon**	1987	Region between Lambarene and N'Djole	NA	5	2
**Liberia**	1970	Grand Geddah	NA	4	0
	2017	Rivercess and Maryland counties	NA	2	0
**Nigeria**	1971	Aba	NA	2	0
	2017	Abia, Akwa Ibom, Bayelsa, Benue, Cross River, Delta, Edo, Ekiti, Enugu, Federal Capital Territory, Lagos, Imo, Nasarawa, Oyo, Rivers	202	88	5
	2018	Abia, Anambra, Bayelsa, Cross River, Delta, Edo, Enugu, Imo, Lagos, Nasarawa, Oyo, Plateau, Rivers	117	49	3
	2019	Akwa Ibom, Anambra, Bayelsa, Cross River, Delta, Edo, Enugu, Imo, Lagos, Oyo, Rivers	98	47	1
	2020	Delta, Lagos, Plateau, Ebonyi, Rivers	35	8	0
	2021	Bayelsa, Cross River, Delta, Edo, Federal Capital Territory, Lagos, Niger, Ogun, Rivers	98	34	0
**Republic of the Congo**	2003	Likouala	NA	11	1
	2009	Likouala	NA	2	0
	2017	Likouala	88	87	6
	2019	Gambona	NA	2	0
**Sierra Leone**	1970	Aguebu	NA	1	0
	2014	Bo	NA	1	1
	2017	Pujehan	NA	1	0
	2019	Kailahun	NA	1	0
	2021	Koinadugu	NA	1	0
**South Sudan** ^¶^	2005	Unity State	9	10	0

**Abbreviation:** NA = not available.

* Includes laboratory-confirmed and probable cases with an epidemiologic (close contact), spatial, or temporal link to a laboratory-confirmed case. For Central African Republic, confirmed cases are in addition to suspected cases; whereas for Nigeria confirmed cases are a subset of suspected cases. For many countries, the number of suspected cases is not available.

^†^ Travel-associated case from Oyo, Nigeria.

^§^ Deaths in the Democratic Republic of the Congo include those among both suspected and confirmed cases.

^¶^ The presence of *Monkeypox virus *in South Sudan was attributed to movement of the virus from the Democratic Republic of the Congo.

**FIGURE:**
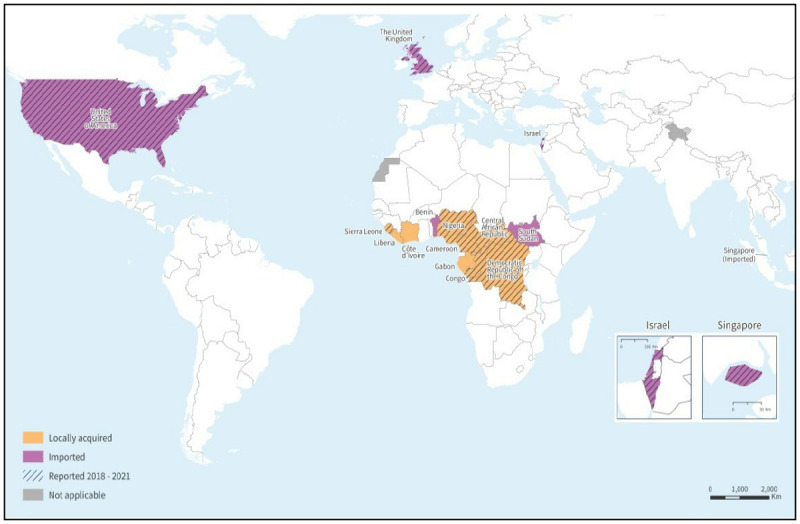
Reported confirmed human mpox cases — worldwide, 1970–2021 **Source:** World Health Organization as of December 6, 2022.

After 39 years without reports, Nigeria experienced a reemergence of cases caused by Clade II beginning in August 2017; this outbreak culminated in May 2018 with 122 confirmed or probable cases among 17 states and included seven deaths (*3*). The country has continued to report mpox cases, with most concentrated in the southernmost states, including in urban settings since the outbreak period. In 2020, during the COVID-19 pandemic, the number of cases reported in Nigeria declined sharply (eight cases reported); however, case reports rose again in 2021. Nigeria has had a number of patients with MPXV and HIV coinfections, including four of the seven fatal cases in 2018. In addition, clinicians noticed atypical presentation that included lesions first appearing on the genitals and the absence of a febrile prodrome (*3*,*4*). Five cases were reported in a prison in 2017, highlighting the need for infection prevention and control in high-density settings, such as correctional facilities and shelters, to prevent person-to-person transmission (*3*).

During 2018–2021, eight independent travel-associated cases of mpox occurred outside Africa in persons traveling from Nigeria (Table 2). The patients were all men aged 30–50 years, and three reported that the rash first appeared in the groin area (*5*–*7*). In one instance, secondary transmission resulted in an infection in a health care provider, and in another instance, in two family members. Each travel-associated case required public health resources to identify community contacts (including airline passengers in some cases) and health care contacts, and to establish care and treatment under strict infection prevention and control measures in health care and some residential environments (*6*).

**TABLE 2:** Reported cases of human mpox outside of Africa,* by country — Israel, Singapore, United Kingdom, and United States, 1970–2021

Country	Year	Location	Confirmed cases	Deaths
Israel	2018	Jerusalem	1	0
Singapore	2019	Central Region	1	0
United Kingdom	2018	Blackpool and Cornwall, England	3^†^	0
	2019	Southwest England	1	0
	2021	Wales	3^§^	0
United States	2003	Illinois, Indiana, Kansas, Missouri, Ohio, Wisconsin	47^¶^	0
	2021	Texas, Maryland	2	0

* All index cases are related to travel from Nigeria except for those in the United States in 2003.

^† ^Includes one secondary transmission in a health care setting in Blackpool from a travel-associated case. https://www.eurosurveillance.org/content/10.2807/1560-7917.ES.2018.23.38.1800509; https://wwwnc.cdc.gov/eid/article/26/4/19-1164_article

^§^ Includes one secondary transmission in a household setting from a travel-associated case, and one tertiary case during isolation of a family member in a health setting with the secondary case. https://www.eurosurveillance.org/content/10.2807/1560-7917.ES.2021.26.32.2100745

^¶^ This outbreak includes confirmed and probable cases and was attributed to an infection of animals in the United States co-housed with a shipment of wild animals from Ghana. https://academic.oup.com/jid/article/194/6/773/864712

In 2021, WHO conducted a survey of orthopoxvirus testing capacity in 127 global laboratories. Among these, 78 (61%) reported working with orthopoxviruses for diagnostic (50), research (52), vaccine development (15), or manufacturing (four) purposes; and 38 (30%) worked with MPXV. Laboratories working with orthopoxviruses were present in the European (30 laboratories), Americas (21), African (11), Eastern Mediterranean (two), Southeast Asian (three), and Western Pacific (11) regions.

## Discussion

MPXV was first identified in 1970 in DRC during the global effort to eradicate smallpox, a disease caused by another *Orthopoxvirus* (*Variola virus*). It was during the period of intensified surveillance for smallpox-like disease in the early 1980s that the clinical presentation, epidemiology, and transmission of mpox were largely defined. It was also during this time that investigations to identify mammalian reservoir species in the regions of Africa with endemic disease (largely in DRC with clade I) occurred. Additional assessments of human disease, including refinements of the different clinical spectrum of illness and epidemiology associated with clade II, occurred during a multistate U.S. outbreak in 2003 associated with the exotic pet trade (*8*). The impact of the route of exposure on disease severity and presentation has been well documented (*8*); however, it was not until the 2017 outbreak in Nigeria when the propensity of clade II MPXV for human-to-human transmission and clinical severity, including death, were recognized (*3*).

Mpox continues to present challenges to public health and health care providers in areas with endemic disease owing to inadequate capacity to diagnose and clinically manage patients and accurately identify exposures. WHO launched an introductory mpox course in 2018 and an intermediate course in December 2021 with content tailored to clinicians and public health providers. The intermediate course offers in-depth information on the epidemiology, presentation, diagnostics, and treatment of mpox and strategies needed for effective prevention and outbreak response, based on information available by late 2021. As of December 14, 2022, 62,196 persons had enrolled in the introductory course and 41,578 had enrolled in the intermediate course.

Until 2017, cases almost exclusively occurred in forested, rural areas where populations might be dependent on wild animal meat for protein. Case-control studies focused on identifying hunting activities that might lead to exposure have not been able to identify a presumptive animal reservoir species. Additional investigations are warranted to examine human interactions with live and dead wild animals, animal meat, and animal products used for cultural, religious, or medicinal purposes. A holistic investigative approach conducted by trained interviewers to identify any risk linked to the diversity of human activities associated with wild animals could be beneficial.

Patients might have difficulty reporting exposures to animals infected with MPXV or to other persons with mpox if exposures are unrecognized. In addition, patients might not provide information regarding possible exposures from sexual interactions if the interactions are associated with stigma or even criminalization. The 2017 outbreak data from Nigeria yielded hypotheses about the role of sexual contact for many of the cases, but investigators were unable to pinpoint this as a significant route of transmission (*3*,*4*). Training of case investigators in stigma-free interview skills and compassionate care might help to better understand mpox transmission, including in historically long-affected areas.

Continued advancements in laboratory diagnostic assays and validation of additional specimen types during the course of infection will aid the ability to detect MPXV, and use of these assays in areas with endemic disease will be necessary to improve surveillance capacity. Laboratory capacity for *Orthopoxvirus *detection has been limited in Africa, hindering the confirmatory diagnosis of suspected mpox disease. Molecular testing (via polymerase chain reaction testing) of lesion specimens has been a standard and effective method of diagnosis of cases in persons with active rash illness. The lack of MPXV-specific serology and existence of cross-reactivity with other orthopoxviruses, including vaccinia virus, hampers the use of serology as a confirmatory test for diagnosis of an infection. This poses challenges for ecologic investigations, where wild mammals must be sampled during an active infection or soon thereafter, as detectable virus and viral DNA appear to be quickly cleared from mucosal surfaces and major organ systems in putative reservoir species (*9*). Further work is needed to better understand the dynamics of disease, including viral dissemination and shedding, in small mammals to guide investigations of animal reservoirs.

Treatment of MPXV infection in patients with immunosuppression due to health conditions is challenging (*4*,*10*). Notwithstanding very limited compassionate use of therapeutics and the launch of some clinical trials and expanded access protocols during the 2022 global outbreak, patient treatment is still dependent on supportive care in both rural and urban areas in many countries (*10*). Research on safe and effective vaccines and therapeutics against orthopoxviruses has been sustained under the smallpox preparedness research agenda mandated and overseen by WHO.** Additional data are needed to assess the efficacy of vaccines and treatments for mpox to develop recommendations and guidance for their use. Such research should be considered in affected countries in partnership with local scientific, and public health authorities. The findings in this report are limited to reporting of cases, which might be incomplete.

Countries with enzootic mpox face an increasingly complex epidemiologic situation, which might include extensive human-to-human transmission, in addition to zoonotic transmission. The use of nonstigmatizing methods for community engagement in populations at risk will be critical to detection and containment. Advances in diagnostics, treatments, and safer vaccines identified through basic and clinical research including during the current outbreak response might be used to improve surveillance, treatment, and prevention of disease in areas where mpox is endemic.

**Summary**

**What is already known about this topic?**

The number of mpox cases reported from rural areas in West and Central Africa had been increasing before 2018.

**What is added by this report?**

During 2018–2021, mpox cases were confirmed in six African countries. Eight primary and three secondary cases associated with travel to Nigeria were identified in four non-African countries. Online training courses on mpox prevention and control have been available since 2018.

**What are the implications for public health practice?**

Mpox continues to present challenges to public health and health care personnel in endemic areas. Improvements in surveillance and community engagement will be critical to detection and containment of the virus. Vaccines and treatments might reduce morbidity and mortality in areas with endemic disease.

## Footnotes

*
https://www.afro.who.int/publications/technical-guidelines-integrated-disease-surveillance-and-response-african-region-third

†
https://openwho.org/courses/monkeypox-introduction

§
https://openwho.org/courses/monkeypox-intermediate
